# One-Year Outcomes of First Metacarpal Osteotomy Using a Precontoured Locking Plate (Shamoji Plate) for Thumb Carpometacarpal Osteoarthritis

**DOI:** 10.7759/cureus.96120

**Published:** 2025-11-05

**Authors:** Shichoh Sonezaki, Hikaru Ogawa, Takahiro Ushijima, Hirotaka Iura, Masaya Kanahori, Hideaki Tanaka, Yosuke Kuroki, Tetsuo Kojima

**Affiliations:** 1 Orthopaedic Surgery, Mizoguchi Hospital, Fukuoka, JPN

**Keywords:** abduction–opposition osteotomy, clinical outcomes, early mobilization, first metacarpal osteotomy, precontoured locking plate, shamoji plate, thumb carpometacarpal osteoarthritis

## Abstract

Introduction: Metacarpal osteotomy is an effective surgical option for thumb carpometacarpal (CMC) osteoarthritis with expanding indications, even in advanced stages. Recently, locking plate fixation has been reported to provide more stable fixation and to allow earlier postoperative mobilization with fewer complications; however, dedicated implants were not previously available, necessitating the intraoperative modification of generic plates. Therefore, we developed a precontoured plate (the 'shamoji plate') specifically designed for the first metacarpal based on 3D CT. This study aimed to determine whether the use of the novel anatomically contoured shamoji plate in first metacarpal abduction-opposition osteotomy would result in significant improvement in clinical findings and radiographic outcomes.

Methods: In this retrospective case series, we included 22 thumbs with Eaton stage 2 or 3 disease that underwent abduction-opposition osteotomy with subsequent fixation using the shamoji plate between January 2022 and May 2024 and had at least 12 months of follow-up. Early range-of-motion exercises were initiated on postoperative days two and three. Clinical and radiographic outcomes were evaluated preoperatively and at the final follow-up.

Results: Almost all clinical metrics significantly improved, including pain, grip strength, lateral pinch strength, thumb range of motion, and the Disabilities of the Arm, Shoulder, and Hand (DASH) scores. Bone union and radiographic realignment (radial and dorsal subluxation correction) were achieved.

Conclusion: The shamoji plate enables safe, early mobilization and reproducible correction without the need for intraoperative plate bending.

## Introduction

Thumb carpometacarpal (CMC) osteoarthritis is a common condition, particularly prevalent in postmenopausal women [1,2]. It has been attributed to morphological differences between women and men [2]. In 1983, Wilson et al. [3] reported the first metacarpal abduction osteotomy for correcting thumb adduction contractures. Subsequently, Pellegrini et al. [4] and Futami et al. [5] proposed variations, including radial extension and abduction-opposition osteotomies, each reporting favorable outcomes despite differences in techniques.

Thumb CMC arthritis has been reported to progress with dorsal subluxation of the first metacarpal in the background of joint laxity [6-8]. In healthy individuals, the metacarpal tilt angle ranges from 79° to 85°, which is less than 90°, and the resultant force vectors tend to act dorsally [9-12]. Patients with thumb CMC arthritis tend to exhibit smaller metacarpal angles than those without, which further facilitates dorsoradial subluxation [4]. The first metacarpal osteotomy aims to correct the metacarpal tilt angle, thereby rebalancing the direction of the muscular force vectors to reduce subluxation and enhance joint stability [4,6]. Additionally, this procedure redistributes joint loading toward the palmar-ulnar cartilage surface, potentially decreasing contact pressure and alleviating pain [4,13].

While traditional methods using K-wires or tension band wiring have shown good clinical outcomes [3,5-7,14-17], they are limited by the need for prolonged postoperative immobilization and have a higher incidence of complications such as pin-site infection and loss of correction at the osteotomy site [7,14,16,18]. Recently, locking plate fixation has garnered attention for enabling stronger fixation and earlier mobilization, with fewer complications [12,13,19-21]. However, dedicated implants were previously unavailable, necessitating intraoperative bending or trimming of the generic plates.

This process is time-consuming and often compromises the locking hole structure or plate strength. Therefore, we developed a precontoured plate specifically designed to fit the first metacarpal bone, and clinical use began in 2022. We hypothesized that the use of the anatomically precontoured 'shamoji plate' in first metacarpal abduction-opposition osteotomy would result in significant clinical and radiographic improvement. Specifically, we investigated short-term outcomes, focusing on correction of radial and dorsal subluxation of the first metacarpal as assessed by standard radiographic parameters.

## Materials and methods

Study population

Thirty patients underwent abduction-opposition osteotomy using the shamoji plate at our institution between January 2022 and May 2024. Patients with at least 12 months of follow-up were included in the study. One patient was excluded due to a protocol deviation (reoperation following early screw breakage). This occurred because the patient did not comply with immediate brace use after surgery and engaged in heavy manual work before suture removal, resulting in screw breakage prior to radiographic union. Other patients were excluded owing to loss of follow-up within one year. Thus, 22 thumbs of 21 patients were evaluated. The study included four males and 17 females, with a median age of 58 years (interquartile range, 50 to 63 years). The dominant hand was affected in 14 thumbs and the non-dominant hand in eight. According to the Eaton classification, seven thumbs were stage 2, and 15 were stage 3.

Implant design

**Figure 1:**
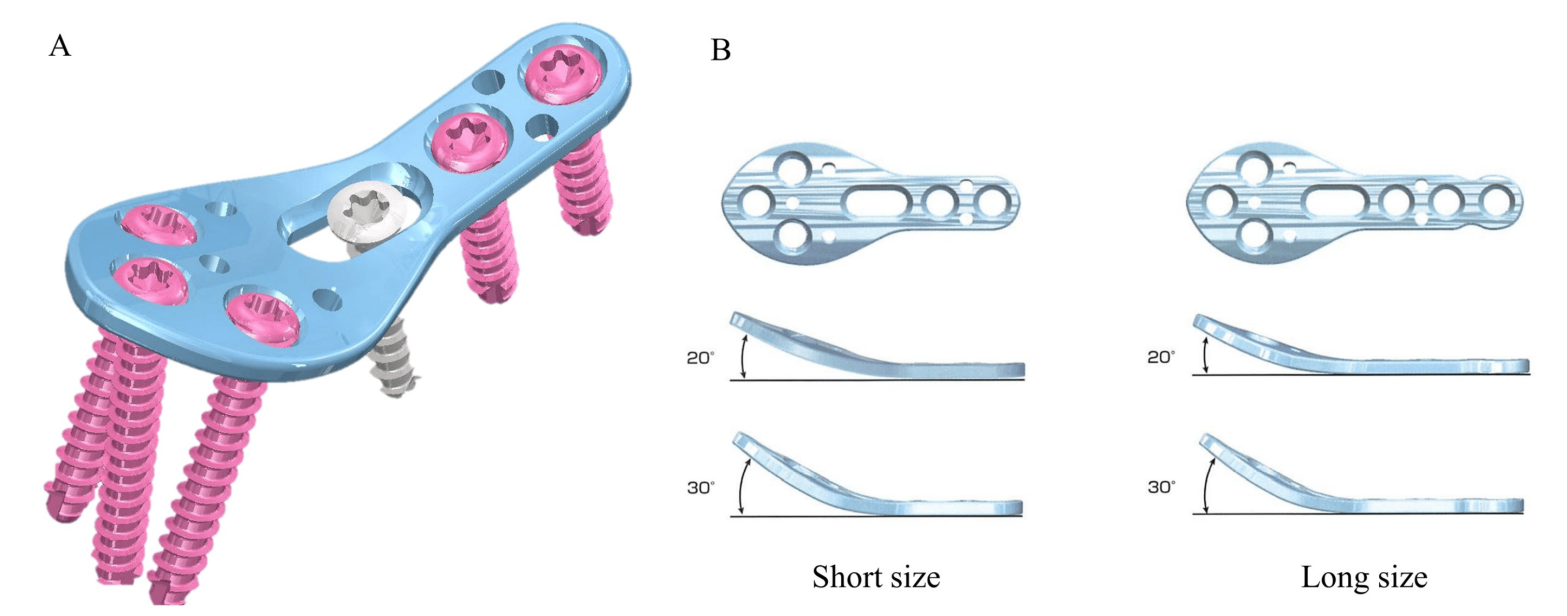
Shamoji plate A: Shamoji plate, B: Variations in size and bending angle Illustration created by Bear Medic Co. Ltd. is used here with permission.

**Figure 2:**
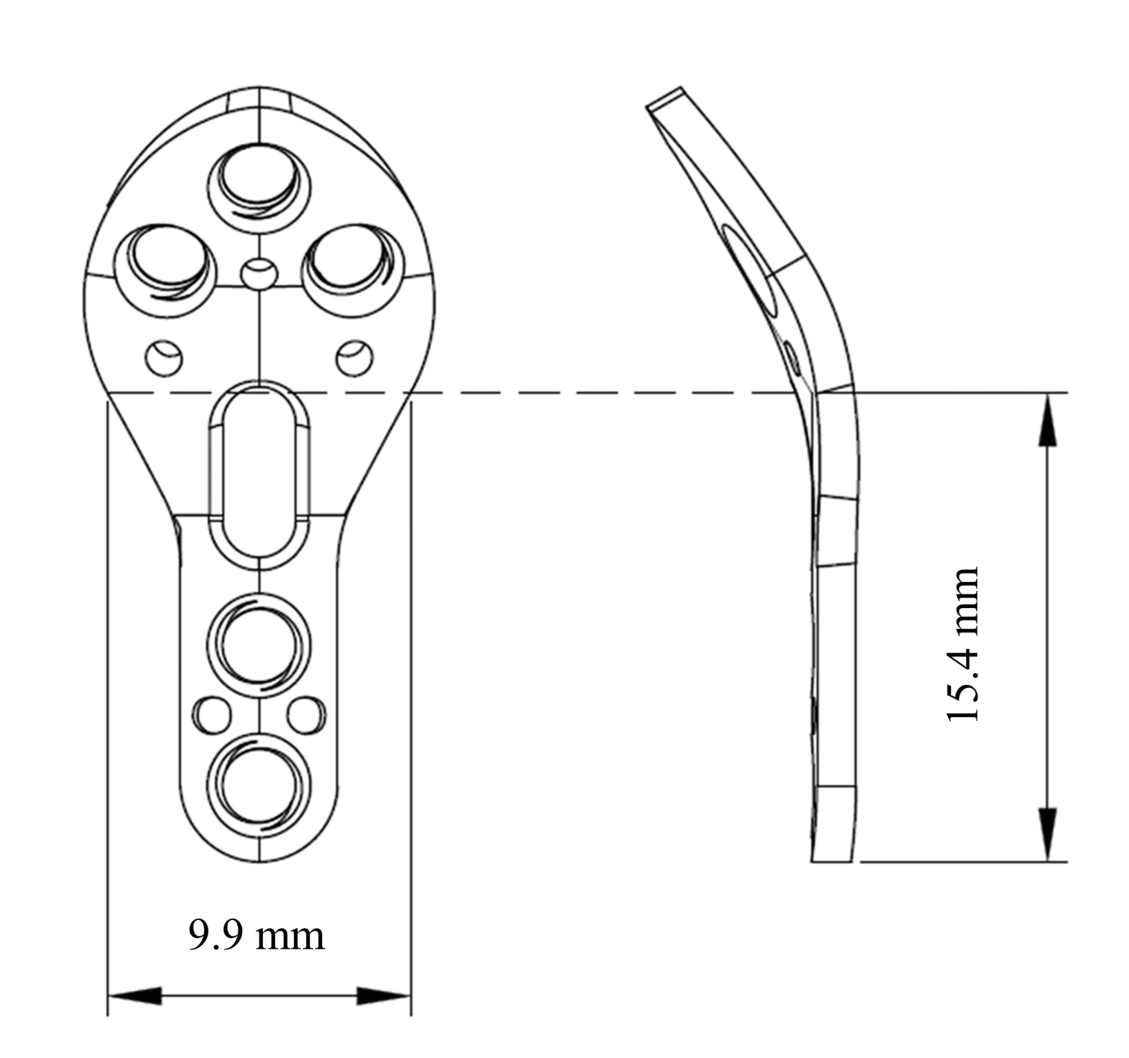
Schematic drawing featuring the dimensions of the curved portion of the plate (short size) Illustration created by Bear Medic Co. Ltd. is used here with permission.

The shamoji plate was designed based on 3D CT data and was anatomically contoured to match the osteotomized first metacarpal bone (Figure 1A). It features a pre-bent configuration at the osteotomy site and is available in 20° and 30° options (Figure 1B). The plate measures 1.3 mm in thickness and is available in two sizes: short and long. The proximal round portion accommodates three locking screws, whereas the distal straight section allows the insertion of three to four screws, including locking and cortical types. A 15° multiaxial locking system allows flexible screw placement. Owing to its resemblance to a traditional Japanese rice paddle (shamoji), we named this implant the 'shamoji plate.' The dimensions of the curved portion of the plate are shown in Figure 2. The bend is located at the most proximal elliptical hole, with a width of 9.9 mm and a height of 15.4 mm (Figure 2).

Surgical technique and rehabilitation

**Figure 3:**
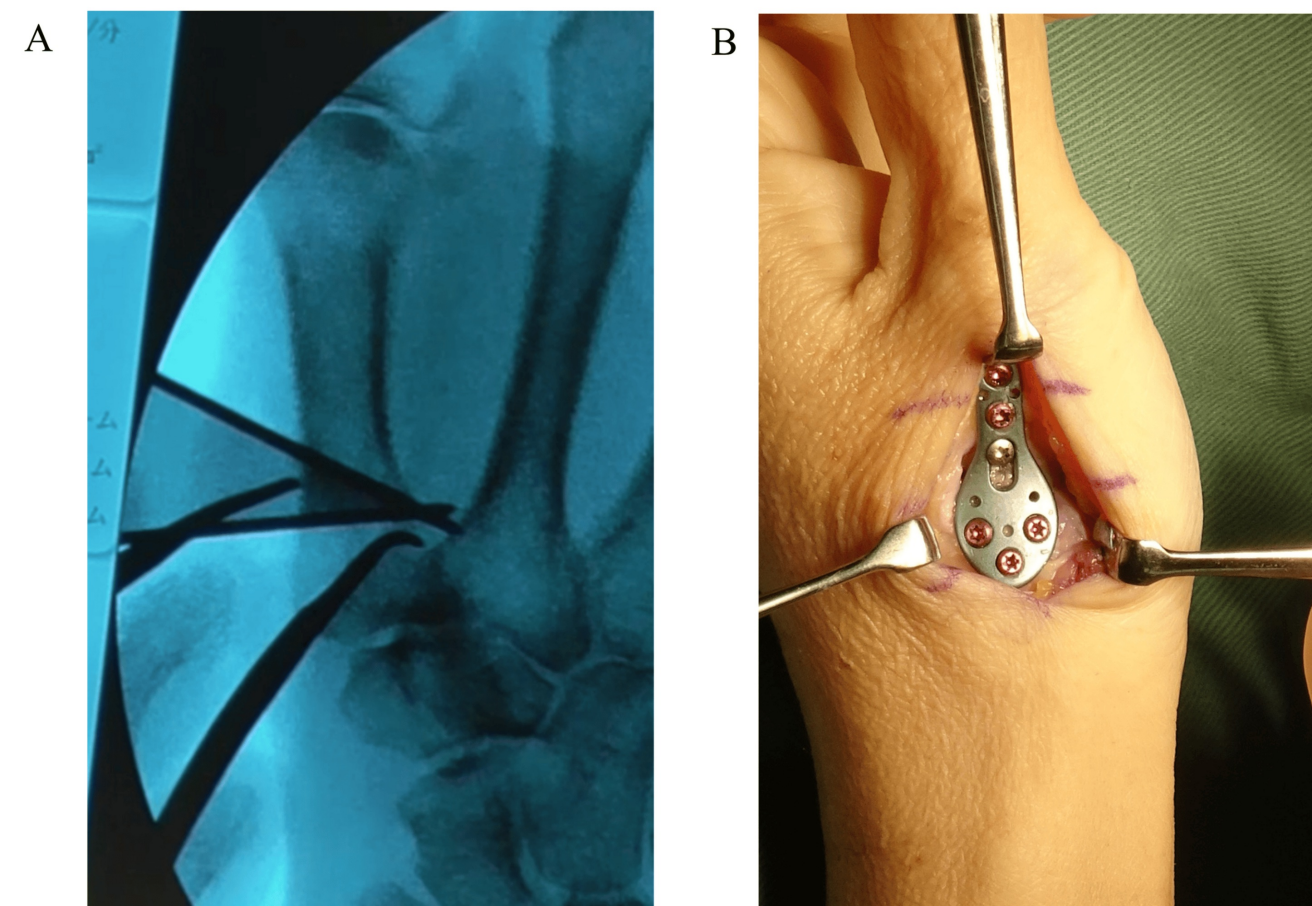
Intraoperative photograph A: Fluoroscopic view showing a K-wire placed parallel to the articular surface, with a second K-wire inserted at a 30° angle using a guide; B: Plate fixation after osteotomy

The patient was placed in the supine position, and an axillary block and general anesthesia were administered. A 4 cm longitudinal incision was made over the dorsal aspect of the first metacarpal, distal to the thumb CMC joint. Care was taken to preserve the sensory branches of the radial nerve. The extensor pollicis brevis tendon was retracted ulnarly. A sharp longitudinal incision was made through the periosteum, and subperiosteal elevation was performed. The osteotomy site was determined using a template as a reference, typically located approximately 12 mm distal to the articular surface. A 1.2 mm K-wire was inserted parallel to the articular surface under fluoroscopic guidance. Slight radial correction was achieved by angling the K-wire from the radial to the ulnar direction, rather than positioning it in a strictly dorsal-to-palmar orientation. Another 1.2 mm K-wire was inserted peripherally at a 30° angle using an angular guide so that it intersects the initial K-wire at the palmar cortex (Figure 3A).

**Figure 4:**
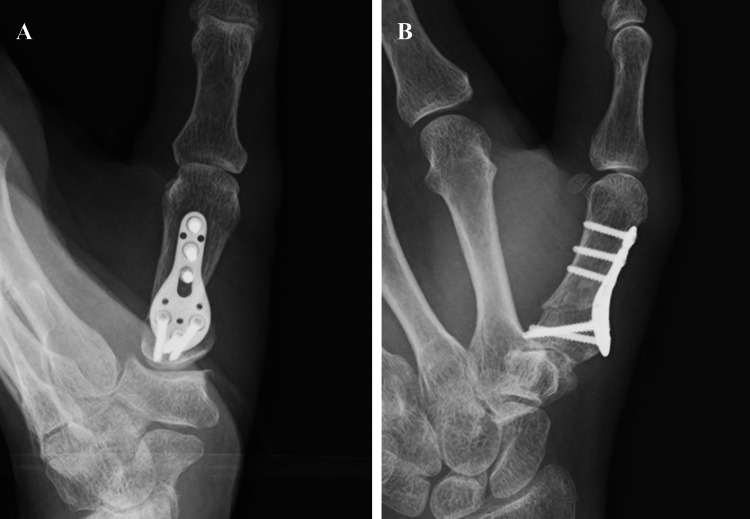
Postoperative radiograph after plate fixation A: Anteroposterior view, B: Lateral view

In cases with a relatively large preoperative metacarpal angle, a smaller correction of 20° was performed instead of 30°, and a 20° shamoji plate was used in a limited number of patients. After cutting the bone with a saw, the wedge fragment was removed. Temporary fixation was performed with a 1.4 mm K-wire, followed by definitive fixation using a shamoji plate and screws. Shamoji plates usually fit well, as they have a pre-bend that eliminates the need for additional bending (Figure 3B). After confirming stable fixation on postoperative radiographs, the periosteum and skin were sutured, and the wound was closed (Figure 4).

A thumb spica cast was applied immediately after surgery, and a splint was substituted following the initial dressing change on the second or third postoperative day, at which time range-of-motion exercises were initiated. During rehabilitation, the splint was removed to allow active exercises of the thumb CMC joint, including palmar and radial abduction. Simultaneously, both active and passive range-of-motion exercises of the interphalangeal (IP) and metacarpophalangeal (MP) joints were initiated, including prevention of adhesion of the extensor pollicis brevis (EPB) tendon. At three weeks postoperatively, the splint was discontinued during rest, and self-directed exercises were encouraged. Gentle passive range-of-motion exercises of the CMC joint were also initiated under supervision. By eight weeks postoperatively, the splint was completely discontinued.

Data collection

Certified hand surgeons and therapists performed all physical assessments. Pain was assessed using a numerical rating scale (NRS) where 0 is for no pain and 10 is for the worst pain, under resting and active conditions. Grip, lateral pinch, and palmar pinch strengths were measured with a dynamometer. The palmar and radial abduction angles of the thumb were measured using a goniometer as the angle between the first and second metacarpals. Patients also completed the Disabilities of the Arm, Shoulder, and Hand (DASH) questionnaire preoperatively and at the final follow-up. The DASH score is a self-administered questionnaire designed to assess upper extremity disability. It consists of 30 items and yields a score ranging from 0 to 100, with higher scores indicating greater disability and more severe symptoms. The validated Japanese version of the DASH questionnaire (Japanese Society for Surgery of the Hand version) was used [22].

Analysis

**Figure 5:**
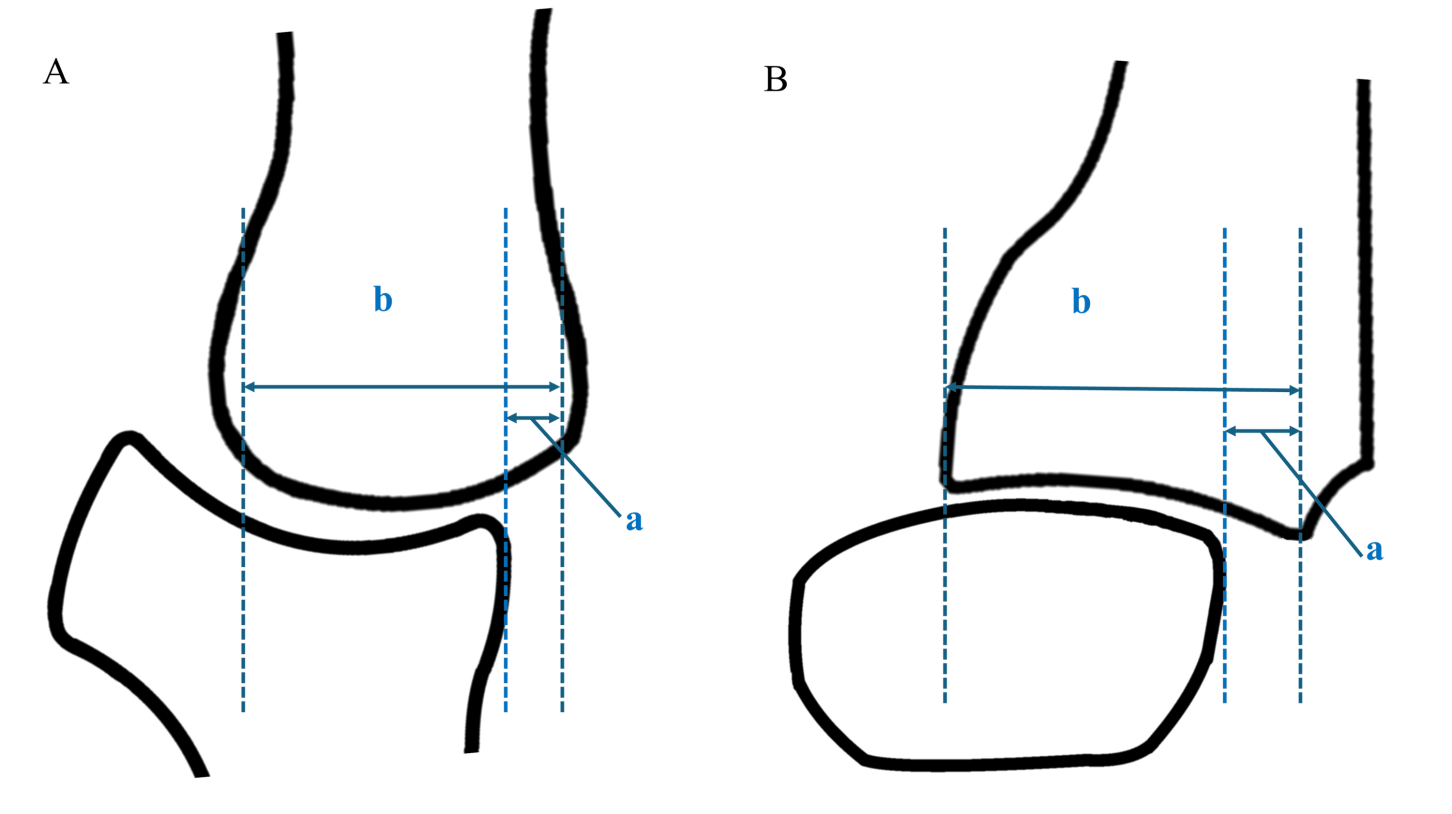
Radial and dorsal subluxation measurements A: Anteroposterior view, B: Lateral view, a: Width of the subluxated joint surface, b: Total width of the joint surface Subluxation ratio=a/b × 100 Original illustration created by the authors

**Figure 6:**
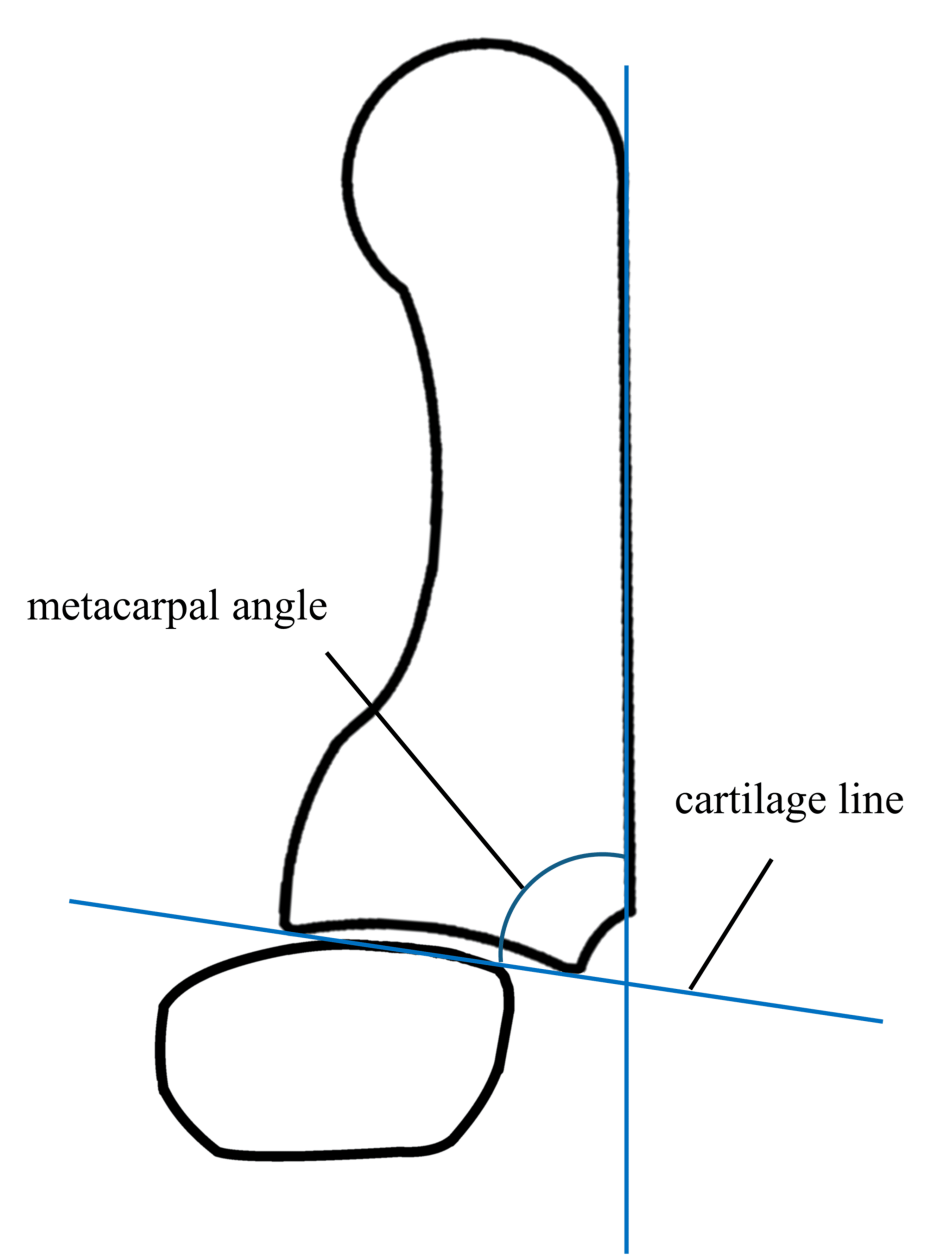
Metacarpal angle is the angle of dorsal cortex and cartilage line Original illustration created by the authors

Preoperative radiographs were used to classify patients’ disease severity according to the Eaton-Glickel classification [23]. Radial subluxation was assessed on anteroposterior radiographs, and dorsal subluxation on lateral views was calculated as the ratio of the displaced joint surface and entire articular length (Figure 5). In the lateral view, the metacarpal angle was defined as the angle between the dorsal cortical line of the first metacarpal and the cartilage line (Figure 6). Additionally, bone union and loss of correction were evaluated with radiographic assessment. Changes in the clinical and radiographic measurements were evaluated using the Wilcoxon signed-rank test. All statistical analyses were performed using JMP® software version 18.0 (SAS Institute Inc., Cary, NC, USA).

## Results

A total of 18 thumbs underwent a 30° osteotomy fixed with a 30° shamoji plate, and four thumbs underwent a 20° osteotomy fixed with a 20° plate. All cases were fixed using the short-size plate. The median follow-up period was 15 months (IQR: 12 to 19 months). Detailed clinical and radiographic outcomes are summarized in Tables 1-2.

**Table 1:** Clinical outcomes Median (IQR); NRS: Numeric rating scale, DASH: Disabilities of the Arm, Shoulder, and Hand

Assessment	Preoperation	Final follow-up	p-value	Effect size
Rest pain (NRS score)	5.0 (0–8.5)	0 (0–0)	<0.001	-0.74
Active pain (NRS score)	8.5 (6.3–10)	2.5 (0–6)	<0.001	-0.79
Grip strength (kg)	11.0 (4–17)	20.8 (16–24)	<0.001	0.71
Lateral pinch strength (kg)	3.3 (2.4–4.5)	4.5 (4.0–5.7)	0.047	0.47
Tip pinch strength (kg)	2.0 (1.7–4.2)	3.4 (2.5–4.1)	0.293	0.27
Palmar abduction angle (°)	45 (30–50)	45 (40–50)	0.012	0.61
Radial abduction angle (°)	35 (25–45)	45 (40–50)	0.007	0.64
DASH score	45.5 (35.8–51.7)	18.2 (4.0–33.9)	<0.001	-0.88

**Table 2:** Radiographic outcomes Median (IQR)

Outcome	Preoperation	Final follow-up	p-value	Effect size
Radial subluxation ratio (%)	8.5 (0–15.1)	0 (0–0)	<0.001	-0.67
Dorsal subluxation ratio (%)	29.5 (21.6–48.8)	14.0 (0–22.5)	<0.001	-0.86
Metacarpal angle (°)	75.5 (72.8–82.0)	95.0 (90.8–100.8)	<0.001	0.88

Significant improvements were observed in resting and activity-related pain (NRS score), radial abduction angles of the thumb, grip strength, lateral pinch strength, and DASH scores. Grip strength improved to 96% (interquartile range (IQR), 90% to 107%), and lateral pinch strength improved to 91% (IQR, 81% to 100%) relative to the contralateral healthy side. However, tip pinch strength did not improve significantly, remaining at 71% (IQR, 63% to 94%) relative to the healthy side. Transient superficial radial nerve symptoms occurred in three cases but resolved by the final follow-up. One patient experienced postoperative backout of a single screw, which caused mild superficial infection due to skin irritation; however, the condition resolved uneventfully after implant removal. No cases of plate breakage or exposure were observed.

Radiographic evaluation confirmed bone union in all patients (median, 74 days; IQR, 56-103), with no cases of correction loss. Radial and dorsal subluxation ratios and the metacarpal angle significantly improved postoperatively compared with the preoperative values. Considering the potential need for future additional surgery, implant removal was performed after bone union in 15 thumbs, excluding seven thumbs in which the patients did not wish to undergo removal.

## Discussion

This study demonstrates that abduction-opposition osteotomy with an anatomically precontoured locking plate specifically designed for the first metacarpal (shamoji plate) achieves reliable one-year clinical and radiographic outcomes through a simplified surgical procedure and a standardized early rehabilitation protocol. Pain, grip, and pinch strength, and radial abduction improved, and alignment was consistently restored. The osteotomy was oriented slightly ulnarly from the dorsopalmar axis, resulting in an angular correction primarily in extension with a minor radial component. This adjustment redirected the first metacarpal’s axial loading vector toward the palmar-ulnar direction, promoting reduction of the subluxation and redistribution of joint contact forces. The shamoji plate provided stable fixation, maintaining the intended correction angle without loss and allowing uneventful bone union in all cases. The relatively long time to radiographic confirmation of bone union was likely because radiographic assessments were performed approximately once a month, which may have delayed the apparent detection of union on imaging. These outcomes were obtained without intraoperative plate bending, which improved the practicality and reproducibility of fixation and facilitated early supervised mobilization. Overall, the results were comparable with those reported in previous plate-based osteotomy studies [12,13,19-21].

Tomaino [17] reported that a 30° extension osteotomy with wire fixation for Eaton stage 1 disease resulted in improvements in pain, joint stability, grip strength, and pinch strength. Similarly, Chou et al. [6] applied the same technique in patients with Eaton stages 1 and 2 and, after a mean follow-up of 9.9 years, found 77% patient satisfaction, with grip strength and range of motion comparable to the contralateral side. Favorable results have also been described in more advanced disease; Gwynne-Jones et al. [15] reported good to excellent outcomes in 22 of 28 thumbs treated with abduction-opposition osteotomy for Eaton stages 2-4.

However, wire fixation provides limited stability and therefore necessitates approximately six weeks of immobilization, during which complications are not uncommon. Pin site infection is the most frequently reported complication [14,18], and wire loosening secondary to deep infection has also been documented [16]. In addition, delayed union, incomplete correction of the preoperative adduction deformity, and loss of correction have been reported [3,7,14,15].

In contrast, locking plates have been increasingly reported to provide stronger fixation and allow earlier mobilization. Shirakawa [19] used the variable angle locking (VAL) hand system to achieve significant improvements in pain, grip strength, and pinch strength while reducing the need for postoperative immobilization to two weeks. Kriechling et al. [21] also reported that using a cutting guide enabled accurate correction (20° extension, 5° adduction), and locking plate fixation prevented complications. Ishibashi et al. [13] performed an extension-abduction osteotomy stabilized with a locking plate (approximately 30° extension and 10° abduction) and reported no implant-related complications while confirming favorable cartilage repair at one year postoperatively. They concluded that plate fixation allows reproducible and stable correction, thereby supporting its role in achieving reliable surgical outcomes. Nevertheless, intraoperative modifications of conventional plates, such as bending a straight plate to fit the desired correction angle, may compromise mechanical strength and potentially increase the risk of loss of correction. In addition, length adjustment often requires trimming of the plate, which makes the procedure more time-consuming.

To further address these issues, the shamoji plate is anatomically contoured to fit the first metacarpal, eliminating the need for intraoperative bending and reducing the risk of plate deformation or loss of locking-hole integrity and reducing between-surgeon variability in achieving the planned correction. In previous reports, the commonly used T-shaped plate had to be bent at its narrower portion to achieve the required contour. In contrast, the shamoji plate has a wider curved section and is precontoured, eliminating the need for intraoperative modification. Its design can therefore be expected to provide higher mechanical strength and more reliable stability. Supporting this, no cases of loss of correction were observed in our series. Thus, the shamoji plate may offer a more reliable and user-friendly option for first metacarpal osteotomy, reducing technical demands and enhancing consistency across surgeons.

While range-of-motion gains with K-wire fixation were limited in the cohort reported by Chou et al. [6], our series showed a significant increase in radial abduction after supervised mobilization beginning as early as postoperative days two and three. Palmar abduction did not change significantly; however, the preoperative median (45°) was already comparable to healthy controls [24] and was preserved postoperatively.

The primary objective of the osteotomy is to correct subluxation and to improve load redistribution, thereby reducing dorsoradial contact stress at the trapeziometacarpal joint. From a pathomechanical standpoint, 3D assessments captured the dorsoradial translation of the first metacarpal during functional tasks [12], while finite-element analyses demonstrated dorsoradial concentration of contact stress at the trapeziometacarpal joint and a reduction in stress when the load vector is redirected volarly/ulnarly [25]. Experimental biomechanics further indicate that ≥15° of extension osteotomy improves stability [26]. These findings support the theoretical rationale that extension or abduction-opposition osteotomy neutralizes the dorsal component of axial loading [12,25,26]. Accordingly, both extension osteotomy [6,7,14,17,18] and abduction-opposition osteotomy [15,16,19] are reasonable strategies, and no clear superiority has been established. However, although the radial component is usually mild, it can be clinically relevant in patients with a shallow trapezial groove; we applied a slight radial correction rather than a pure sagittal extension. Nevertheless, because excessive radial correction may compromise the intended extension or worsen plate fit, overcorrection was avoided.

With respect to the correction angle, most studies recommend a 30° osteotomy [4-6,16,17], which is approximately equivalent to Wilson’s 5-mm wedge osteotomy. Parker et al. [7] reported good, long-term results with a 15° extension osteotomy; however, their study included only eight patients, with only two with Eaton stage 3 disease. A biomechanical study by Koff et al. [26] reported that a 15° extension osteotomy improved joint stability more effectively than a 10° extension osteotomy, indicating superior stability at 15°; however, higher angles have not been thoroughly investigated. Keller et al. [12] focused on the mismatch between the anatomical and functional axes and suggested that an average correction of 22.6° (1 cm from the joint surface) or 26.7° (1.5 cm) is required. Honigmann et al. [20] also applied a 15° to 30° extension osteotomy depending on the case and added ligament reconstruction in patients with residual instability. On the other hand, Shirakawa [19] reported that greater extension angles were associated with a better reduction in Eaton stage 3 cases. Smaller correction angles may be sufficient for early-stage disease or when the metacarpal angle is preserved. In our protocol, the correction angle was determined intraoperatively using K-wires and angle templates, and fixation was performed with a precontoured shamoji plate according to the surgeon-specified angle. This eliminated the need for intraoperative plate bending and enabled standardized fixation using a multiaxial locking mechanism.

At our institution, most cases involved advanced disease; therefore, a 30° osteotomy was generally used as the standard, whereas a 20° osteotomy was applied in milder cases with relatively large preoperative metacarpal angles. Even with a uniform 30° osteotomy, thumb shortening averaged approximately 3 mm, differing by only 1 mm to 1.5 mm compared with 15° or 20° osteotomies. Thus, joint centralization was prioritized over thumb shortening. Because patients with thumb CMC osteoarthritis often present with ligamentous laxity, achieving a slightly overcorrected alignment beyond 90° of the metacarpal angle is considered desirable. However, excessive correction may lead to notable thumb shortening or articular incongruity; therefore, correction angles greater than 30° are generally not recommended.

This study has some limitations. Its retrospective design may have introduced inherent bias, and the radiographic evaluations were based on plain radiographs. Moreover, the follow-up period was short. Although plate fixation for first metacarpal osteotomy is generally considered to provide stronger stability, allow earlier mobilization, and reduce complications such as infection or displacement compared with pin fixation, this study did not include a direct comparison between the two fixation methods. Further studies with longer follow-up and comparative analyses, including 3D evaluation, are warranted.

## Conclusions

Rigid fixation with a precontoured locking shamoji plate shortened the postoperative immobilization period and enabled an earlier, more comfortable return to daily activities. Improvements were demonstrated in pain, range of motion, DASH scores, and radiographic alignment. Designed specifically for the first metacarpal, the plate provided an anatomic fit and eliminated the need for intraoperative plate bending. These findings support its clinical utility for first metacarpal osteotomy. Its applicability may extend beyond Japanese patients, given that body size and skeletal anatomy are comparable.
